# Fiber Amplifiers and Fiber Lasers Based on Stimulated Raman Scattering: A Review

**DOI:** 10.3390/mi11030247

**Published:** 2020-02-26

**Authors:** Luigi Sirleto, Maria Antonietta Ferrara

**Affiliations:** National Research Council (CNR), Institute of Applied Sciences and Intelligent Systems, Via Pietro Castellino 111, 80131 Naples, Italy; antonella.ferrara@na.imm.cnr.it

**Keywords:** stimulated raman scattering, fiber optics, amplifiers, lasers, optical communication systems

## Abstract

Nowadays, in fiber optic communications the growing demand in terms of transmission capacity has been fulfilling the entire spectral band of the erbium-doped fiber amplifiers (EDFAs). This dramatic increase in bandwidth rules out the use of EDFAs, leaving fiber Raman amplifiers (FRAs) as the key devices for future amplification requirements. On the other hand, in the field of high-power fiber lasers, a very attractive option is provided by fiber Raman lasers (FRLs), due to their high output power, high efficiency and broad gain bandwidth, covering almost the entire near-infrared region. This paper reviews the challenges, achievements and perspectives of both fiber Raman amplifier and fiber Raman laser. They are enabling technologies for implementation of high-capacity optical communication systems and for the realization of high power fiber lasers, respectively.

## 1. Introduction

Optical communication systems require optoelectronic devices, such as sources, detectors and so on, and utilize fiber optics to transmit the light carrying the signals impressed by modulators. Optical fibers are affected by chromatic dispersion, losses, and nonlinearity. Dispersion control is, usually, achieved via fiber geometry and material composition. Losses limit the transmission distance in modern long haul fiber-optic communication systems, so in order to boost a weak signal, optical amplifiers have been developed. The basic idea, behind the fiber amplifier realisation, was to allow to the signal to remain in optical form throughout a link or network. Fiber amplifiers as repeaters offer a number of advantages, including the ability to change system data rates, or to simultaneously transmit multiple rates—all without the need to modify the transmission channel. The most important advantage is that signal power at multiple wavelengths can be simultaneously boosted by a single amplifier—a task that would require a separate electronic repeater for each wavelength. The state-of-the-art of optical amplifiers are erbium-doped fiber amplifiers (EDFAs), which are formed by doping the glass fiber host with erbium ions. In EDFAs, gain by stimulated emission at wavelengths in the vicinity of 1.55 µm is obtained, by optical pumping using light at either 1.48-µm or 0.98-µm wavelengths. EDFAs are lumped amplifier, this means that the signal, monotonically attenuated in the fiber span, is amplified at a point of the EDFA location to recover the original level, before entering the next fiber span. The distance between amplifiers is determined by the span loss, by the limit imposed from the power maximum value allowed in the fiber, without inducing nonlinear effects, and by the minimum acceptable power, avoiding a degradation of the signal-to-noise-ratio. EDFAs are compact and highly efficient devices with high gain and low noise [1].

Optical multiplexing allows to combine multiple optical signals into one to make full use of the immense bandwidth potential of an optical cannel. The basic idea is to divide the huge bandwidth of optical fiber into individual channels of lower bandwidth, so that multiple access with lower-speed electronics is achieved. The large gain bandwidth of EDFAs has enabled the realization of dense wavelength-division multiplexed systems, in which terabit/sec data rates have been demonstrated. EDFAs operate in the C-band (from 1530 to 1565 nm) while extended-band EDFAs can provide gain in the L-band (from 1565 to 1625 nm). However, the capacity of most fibers can be extended by opening up the S band (from 1480 to 1530 nm), and the S+ band (from 1430 to 1480 nm). In addition, we note that earlier transmission systems were deployed in the 1310-nm band, which can stretch between 1280 and 1340 nm, while there could be also a 1400-nm band, which is only useful in new fibers using special drying techniques to reduce the water peak absorption around 1390 nm. Definitely, the available communication range could span from 1270 to 1650 nm, corresponding to about 50 THz bandwidth, but the current strong limitation is that a great part of this range is inaccessible by EDFAs [1,2,3,4].

In optical communications, fiber nonlinearities are the basis of a number of devices such as amplifiers, switching and logic elements. The nonlinear effects in optical fiber can be divided in two class. The first one is due to the Kerr-effect, i.e., intensity dependence of refractive index of the medium, which manifests itself in three different effects: Self-Phase Modulation (SPM), Cross-Phase Modulation (CPM) and Four-Wave Mixing (FWM), depending upon the type of input signal. The second one is due to inelastic-scattering phenomenon, in which the optical field transfers part of its energy to the nonlinear medium. Such an inelastic-scattering phenomenon can induce stimulated effects such as Stimulated Brillouin-Scattering (SBS) and Stimulated Raman-Scattering (SRS). We note that each type of stimulated scattering process can be used as a source of gain in the fiber. In both phenomena, at high power level, the intensity of scattered light grows exponentially if the incident power exceeds a certain threshold value. Raman amplification is more useful, because of the relatively large frequency shift and the broader-gain bandwidth. The basic difference between them is that in the case of Brillouin the interaction is between the guided optical wave and low-frequency acoustic phonons, while in the case of Raman the interaction is between the guided wave and high-frequency optical phonons. Another fundamental difference is that SBS in optical fibers occurs only in the backward direction whereas SRS can occur in both directions [2,3,4].

In long-range high-datarate systems, involving high optical power levels and signals at multiple wavelengths, the consequences of fiber nonlinearities can be: (1) the generation of additional signal bandwidth within a given channel, (2) modifications of the phase and shape of pulses and can cause spectral broadening, which leads to increased dispersion, (3) the generation of light at other wavelengths at the expense of power in the original signal, and (4) crosstalk between signals at different wavelengths and polarizations. The first two arise from self-phase modulation, while the third and fourth effects arise from stimulated Raman or Brillouin scattering or four-wave mixing. These last can be used when generation or amplification of additional wavelengths is desired. We note that in order to obtain an error-free system performance, due to the finite sensitivity of the optical receiver, the signal should have a high-enough level. On the other hand, the nonlinear effects are proportional to the product of the signal power, P, and the transmission distance L. This means that by increasing the signal level, the distance and the transmission bit rate (speed), all the problems—dispersion, noise, and nonlinearity in the fiber are increased, too. It is worth noting that the signal distortions is an issue when the nonlinearity is involved, because it can couple a number of detrimental effects together, such as dispersion, noise, polarization mode dispersion, polarization-dependent loss/gain, etc. [2,3,4].

SRS is a nonlinear process, observed for the first time in 1962, which lies at the heart of fiber Raman amplifiers and fiber Raman lasers (FRAs and FRLs). Direct fiber optical amplification obtained by SRS, called Raman amplification, was demonstrated in the early 1970s. In Raman amplification, a power transfer from pump (s) to information carrying signals (usually described as probes) can occurs, if there is a sufficient pump power within the fiber. In principle, when optical pump energy is added along with signals in ordinary optical fibers, optical amplification can take place, providing low-noise, flat and wideband signal gain. The more attractive advantage is that FRAs, operating in a signal band outside the EDFA bands, could open new transmission windows in the future [2,3,4].

High-power fiber lasers have had a significant development, achieving output powers of multiple kilowatts from a single fiber. Due to its inherent material advantages, Ytterbium has been the primary rare-earth-doped gain medium, so fiber lasers are largely confined to its narrow emission wavelength region. It is worth noting that because of its Raman-shifted out, SRS is a workable method for generating coherent radiation at new frequencies. Taking advantages of the technology of high-power lasers, which serve as their input, FRLs can lead to conversion to wavelengths higher than the starting wavelength, using a series of Raman–Stokes shifts. In fiber-lasers, the tight spatial confinement of pump light, which is maintained over relatively large distances, significantly lowers threshold pump powers down to practical levels and enables continuous-wave operation. The main advantage of Raman laser is that essentially any laser wavelength can be achieved from the ultraviolet to the infrared with a suitable choice of the pump wavelength, providing that wavelengths are within the transparency region of the material and sufficiently high nonlinearity and/or optical intensity are reached. For this reason, currently, FRLs are the only wavelength scalable, high-power fiber laser technology that can span the wavelength spectrum [2,3,4].

Due to the extent of subject, a comprehensive review including mathematical [5,6,7,8] and physics aspects of SRS [9,10,11,12,13,14], architecture of fiber system [15,16,17,18,19] and laser systems would be impossible to realize. Therefore, the aim of our paper is to provide an overview of the field, emphasizing physical effects and working principles of fiber optics amplifier and laser based on SRS. This approach could have the advantage to provide a quick look of the state of art and to allow to scientists, who are new to Raman amplification, to go into the field. In addition, a comprehensive list of references is also provided for readers who wish to pursue any of the topics in more detail.

The paper is organized as follows. In next paragraph, for the sake of completeness an essential theoretical background is reported. In Section 3, Raman amplifiers are described, which are generally divided into two categories, namely, distributed and lumped amplifiers. Of course, the two classes can be combined to form a hybrid amplifier, where the discrete FRA can be replaced by an EDFA [20]. In Section 4, Raman lasers in fiber are introduced, which can be classified into two general categories. In the former, the wavelength is shifted by one Raman–Stokes shift, while, in the latter, called cascaded Raman laser, the wavelength is shifted by multiple Raman–Stokes shifts. In the last section, a brief introduction about Raman soliton laser is reported, too.

## 2. Theoretical Background

**Figure 1:**
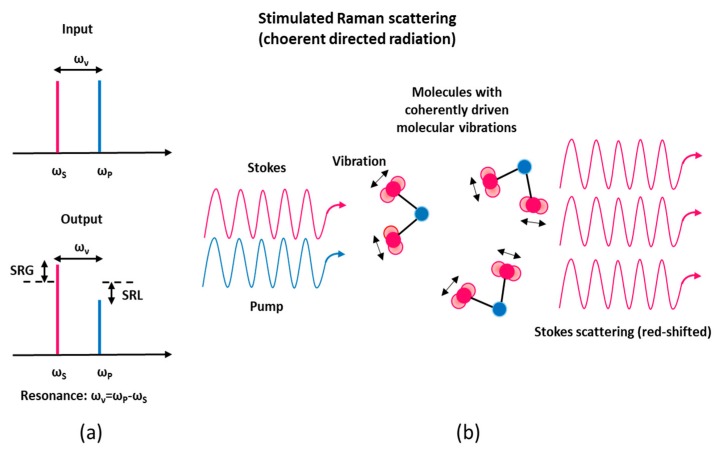
Stimulated Raman-Scattering (SRS) principle. (**a**) Pump–probe modalities associated with the SRS process are pointed out: SRG, stimulated Raman gain; SRL, stimulated Raman loss. (**b**) Stimulated Raman scattering occurs through inelastic scattering of probe photons off from vibrationally excited molecules that interfere coherently.

SRS can be obtained by irradiating a sample with two simultaneous light sources: a light wave at frequency *ω_L_* (the pump laser wave) and a light wave at frequency *ω_S_* = *ω_L_* − *ω_υ_* (the Stokes Raman wave), where *ħω_υ_* corresponds to a vibrational energy. In SRS phenomenon, when the frequency difference between the pump and the Stokes laser beams matches a given molecular vibrational frequency of the sample under test, there is a transfer of energy from the high power pump beam to the probe beam, which can be co-propagating or counter-propagating. The SRS effect occurs in the form of a gain for the Stokes beam power (stimulated Raman gain, SRG) and a loss for the pump beam power (stimulated Raman loss, SRL), see Figure 1a. It is worth noting that in SRS, because of its coherent nature, the molecular bonds oscillate with a constant phase relation (see Figure 1b) and interfere constructively within a certain macroscopic area (e.g., inside the focus area of laser beam), therefore the SRS signal can be orders of magnitude more sensitive than spontaneous Raman scattering [9,10,11,12].

**Figure 2:**
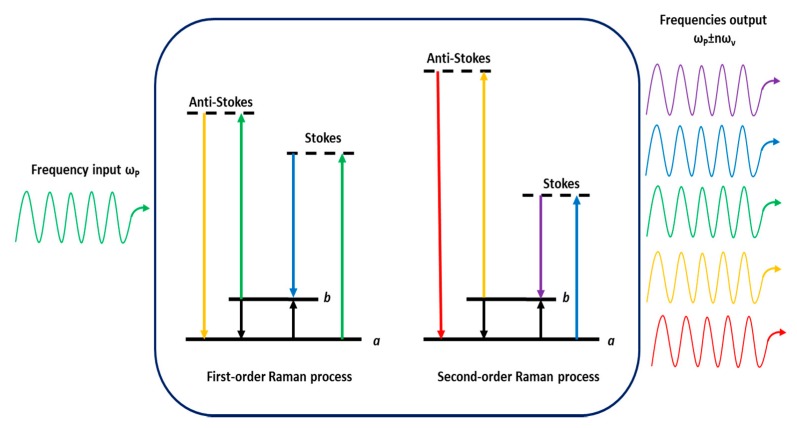
Cascaded Raman scattering principle. An input frequency *ω_P_* (green) can cause the spontaneous emission of frequencies *ω_P_* ± *ω_ν_,* [Stokes (blue) and anti-Stokes (orange)]. This initial spontaneous event provides the seed photons necessary for subsequent first-order SRS (**left**). The first-order Stokes and anti-Stokes photons then can cause second-order Raman scattering (**right**) to produce new frequencies *ω_P_* ± 2*ω_ν_* (red and violet) and so on through propagation, resulting in a broadband ladder of frequencies *ω_P_* ± *nω_ν_*.

In SRS, high-order Raman sidebands can be produced by a field propagating through a medium optically polarizable. Many Stokes frequencies can be generated at the output when the pump power exceeds the threshold value, thus Stokes frequencies at *ω_P_* − *ω_ν_*, *ω_P_* − 2*ω_ν_* and of anti-Stokes frequencies at *ω_P_* + *ω_ν_*, *ω_P_* + *2ω_ν_* can be observed. If the intensity of the first-Stokes wave is enough high, it can generate a ‘‘second-Stokes’’ beam (see Figure 2). By iterating this process, higher Stokes order can be generated, leading to the so-called ‘‘cascaded’’ SRS. The intensity of the *i*th Stokes order thus depends on the conversion rate from the (*i* − 1)th Stokes order (proportional to *g·I_i_·I*_*i*−1_) and loss rate to the (*i* + 1)th order (proportional to *g*·*I_i_·I*_*i*+1_) where *g* is the Raman gain of material. We note that frequency conversion can be obtained over a wide range of output wavelengths (visible and UV) within a single Raman laser system. For example, it is possible to use standard diode-pumped crystalline solid-state lasers to generate several Stokes order by cascading effect [9,10,11,12,13,14].

Optical fibers are an excellent medium for utilizing SRS. There are two key parameters for performances optimization of Raman fiber amplifier and laser. The first, linear losses are fundamentals for enabling long interaction lengths. We note that different types of fibers have different low-loss wavelength windows. For example, the silica fibers, which are used in the majority of optical fiber applications, have extremely low losses (<1dB/km) in the near-IR region, whereas they are very lossy in the mid-IR region. The second, effective area: Because of their small core diameter <10 μm, single-mode fiber has the ability to confine light to small mode areas, which significantly enhances nonlinearity. We note that in silica glass, in spite of intrinsically small values of the third-order nonlinear coefficients, which are smaller by a factor of 100 or more compared to many crystals and liquids, nonlinear effects in optical fibers can be observed at relatively low power levels.

In the past century, fused silica has been the main material used for long and short-haul transmission of optical signals, because of its good optical properties and attractive figure of merit (trade-off between Raman gain and losses). Since in amorphous materials, molecular vibrational frequencies spread out into bands that overlap and create a continuum, in silica fibers, the Raman gain (g_R_(Ω)) spectrum extends over a large frequency range (up to 40 Thz). The maximum of the Raman gain spectrum in silica fibers is downshifted from the pump frequency by about 13.2 THz (440 cm^−1^), corresponding to 100 nm in the telecom window. Therefore, being the signal wavelength usually around 1550 nm, the pump light wavelength has to be about 1450 nm. We note that at high enough powers there can be lasing at all frequencies with sufficient Raman gain [21,22,23,24,25,26,27], though the peak gain is at a specific frequency shift. The following fundamental properties of Raman gain are critical for designing Raman fiber amplifiers and lasers:

(1) Raman gain has a spectral shape that depends primarily on the frequency separation between a pump and signal, not on their absolute frequencies. This follows from energy conservation: Their frequency separation must be equal to the frequency of the created optical phonon;

(2) Since Raman gain does not depend on the relative direction of propagation of pump and signal, SRS occurs almost uniformly for all the orientations between the pump and signal propagation direction, as a consequence FRAs can work for both the copropagating, counter-propagating or bidirectional pumps with respect to the signal;

(3) SRS is a fast process, response time of fused Silica is evaluated to be less than 100 fs [28,29]. For most applications, this appears instantaneous, and especially in relation to the application as Raman amplifiers in optical communications systems;

(4) Raman gain is polarization dependent. Raman gain coefficients are usually quoted for parallel linearly polarized pump and Stokes fields. For perpendicular polarizations the gain is more than an order of magnitude smaller than the parallel one. In typical long fibers, which do not maintain linear polarization, the Raman gain will assume some average value which is approximately half of the polarized gain [30,31];

(5) Peak Raman gain in a specific fiber can be different based upon the material composition. For example, in phosphosilicate fiber the peak gain is at 1330 cm^−1^. This larger Raman shift is attractive for many applications, since fewer Stokes shifts are needed to reach the final desired wavelength.

Concerning materials, two problems are of great interest: (1) Development of glasses with a higher Raman gain coefficient; (2) increase of Raman gain bandwidth [32,33,34,35,36]. The increase of dopant content in Ge- and P-doped silica fibers with a simultaneous reduction of optical losses seems the most straightforward solution to the first problem. The second option is to use multicomponent glasses, including heavy-metal oxides-doped glasses, which also permits the development of glasses with the Raman gain bandwidth of several hundreds of cm^−1^ [37,38,39,40,41,42]. However, in multicomponent glass approach the current limitation is to obtain low-loss fibers.

In fiber communication systems, two basic issues are related to SRS. First, pump-to-Stokes coupling provides a mechanism for crosstalk from short- to long-wavelength channels, which is more efficiently when the channel frequency spacing is close to the one associated with the maximum Raman gain [43]. In wavelength division multiplexed (WDM) systems, within the 1.53- to 1.56-µm erbium-doped fiber amplifier window, channel spacings on the order of 100 GHz are used, thus due to the Raman gain peak at approximately 500 cm^−1^, Raman gain is considerably reduced, but is still sufficient to cause appreciable crosstalk, which can lead to system penalties depending on the number of channels [44]. Second, the conversion to Stokes power from the original signal—a mechanism by which signal power can be depleted. A related problem is walkoff [45] occurring between the signal and Stokes pulses, since these will have different group delays. If pulses are of sub-picosecond widths, additional complications arise due to the increased importance of SPM and cross-phase modulation (XPM) [6].

## 3. Fiber Raman Amplifiers (FRAs)

SRS in a pulsed mode was observed, for the first time, in a liquid core fiber by Ippen in 1970 [46]. The first SRS observation in a single-mode fiber (SMF) was made by Stolen et al. in 1971 [47], whereas the first SRS observation in continuous wave (CW) mode was obtained by Stone [48] in a hollow fused silica fibers filled with benzene and with a pump power value less than 100 mW. All these authors pointed out the fiber geometry allows SRS amplification/generation, due to the confinement of high power pump power density over long distances [49].

The fundamental advantages of FRAs are:

(1) They do not require special dopants. This means that ordinary, transparent, passive optical fibers can be turned into a Raman amplifier;

(2) Raman gain is obtainable in any conventional transmission fiber, which can be used as transmission line and as Raman gain medium, too. Therefore, Raman amplification is compatible with most available transmission systems;

(3) Raman amplification can be provided at any wavelength. Being the Raman gain non-resonant, it is available over the entire transparency region of fiber ranging from approximately 0.3 to 2 micron, if the appropriate pump sources are available.

(4) The broad gain bandwidth, obtained in conventional transmission fibers, is an important property for WDM systems.

(5) Raman gain has a high-speed response. Therefore, in principle, in fiber the entire bit stream can be amplified without any distortion in high bit rate systems.

### 3.1. Distributed Fiber Raman Amplifiers

In a distributed FRA, pump at the optical frequency *ω_P_* and signal at the frequency *ω*_s_ = *ω_P_* − *ω_ν_* are jointly launched into the same ordinary optical fiber. Raman optical amplification takes place when their frequency difference Ω = *ω_P_* − *ω*_s_ lies within the bandwidth of the Raman-gain spectrum. The distributed FRA guides light at both the signal and pump wavelengths and it is normally single mode to ensure the best overlap of all traveling waves. A distributed FRA unit is a pumping unit at 14xx nm without gain medium and it, usually, consists of pump lasers, optical couplers to combine them, isolators and a few monitor taps, as well as the WDM coupler for signal and pump. Depolarizers are necessary when the pump is polarized, in order to suppress the polarization dependence of Raman amplification.

**Figure 3:**
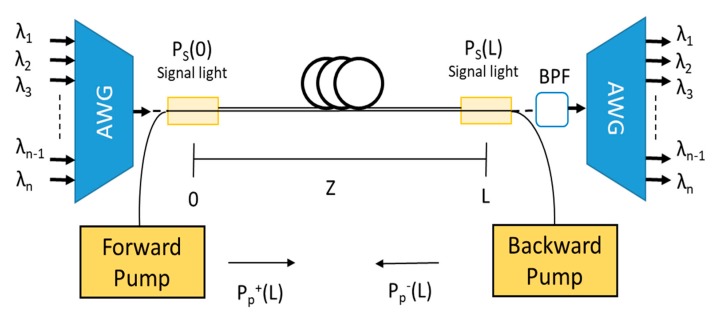
Schematic representation of multiplexing/demultiplexing based distributed optical Raman amplifier.

Figure 3 shows a configuration in which pumping light propagates bi-directionally in distributed FRA. Moreover, the system is provided with band pass filter (BPF) and arrayed waveguide grating (AWG) devices which act as demultiplexing unit in the receiving side.

As mentioned before, in a distributed FRA both forward and backward pumping schemes are possible. In the forward pumping, the signal is amplified near its fiber-input end, where the signal power is relatively high. Thus, this scheme corresponds to a booster amplifier. In the backward pumping, the optical signal is amplified near its fiber-output end before photodetection, where the signal power may be so weak as to be comparable to the noise light power. Since this scheme is used to improve detectivity or to reduce the minimum detectable signal power, it can be considered as a detection preamplifier [50,51].

We note that the process of Raman amplification takes place so rapidly that, unless the intensity noise of the forward pumping light is sufficiently small, the pumping light noise will be transferred to the signal light resulting in increasing transmission bit error rates. Since signals propagating in the opposite direction to the pump will average over the fluctuations, in order to mitigate unwanted crosstalk between the pump and the signal, in many cases only backward pumping is used [52,53]. Typically, high-powered counter propagating Raman pumps are deployed in conjunction with discrete amplifiers, such as EDFAs.

**Figure 4:**
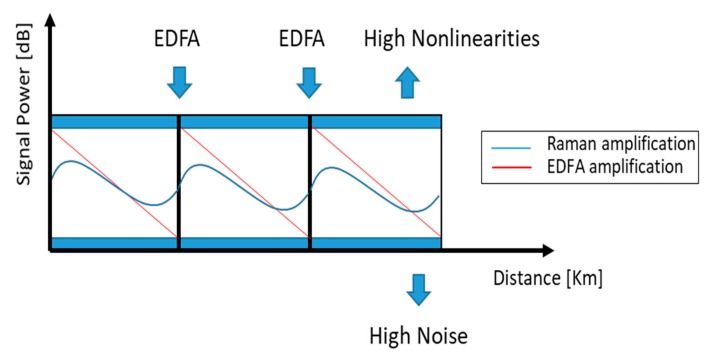
Ideal distributed amplifier: the loss is counterbalanced at every point along the span, leading to an improvement of the signal to noise ratio (SNR) respect to (ideal) discrete amplification.

Distributed FRAs offer several advantages: simplicity, because they provide direct optical amplification of signal light in the transmission fiber; flexibility in the use of signal wavelengths, because the Raman gain peak is dependent on the pump wavelength and not the emission cross section of a dopant; broad gain bandwidth, which allows to employ multiple pumps. However, one of the major benefits, only obtainable through the use of distributed amplification, is that signal gain may be pushed into a transmission span preventing the signal from decaying as much as it otherwise would have if no amplification was provided within the span. As a consequence, the signal-to-noise ratio does not drop as much as it would have in a system based on transmission through a passive fiber followed by a discrete amplifier (see Figure 4). Thus, lower signal powers can be used, nonlinear penalty are reduced and higher loss can be tolerated. This improved noise performance may be used in different ways. One way is to extend the reach between repeaters, another is to extend the total reach of a transmission system, and a third one is to improve the transmission capacity.

Using distributed FRAs, the transmission fiber becomes an amplifier, giving an important advantage but, at the same time, the optimization of the fiber design with respect to amplifier performance becomes more difficult due to the need to optimize the fiber for signal transmission, too. Several parameters have to be taken into account to evaluate the performance of a distributed FRAs, such as linear noise accumulation, pulse distortion due to group velocity dispersion, effects due to Rayleigh scattering, nonlinear interactions between pump and signal channels, and pump–signal crosstalk. Considering that in distributed amplifiers the gain is gathered over tens of kilometers and that the Raman effect is a very fast process (response time in the order of femtoseconds), these noise sources become significant.

In Ref. [54] a 25.6 Tb/s experiment was demonstrated over a 240 km repeated span by employing 160 WDM channels with 50 GHz channel spacing in the C+L bands. The authors used backward Raman pumping to increase the received optical signal to noise ratio (OSNR) and simplify the optical repeaters, while an optical equalizer was used in the receiver to minimize channel distortion caused by narrow optical filtering.

### 3.2. Discrete Fiber Raman Amplifiers

**Figure 5:**
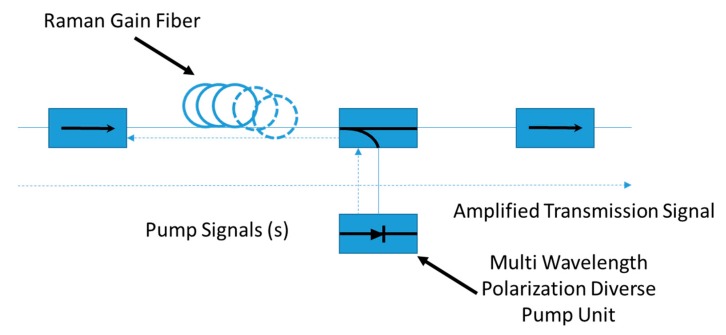
Discrete fiber Raman amplifier (FRA) scheme with pump power confined to the lumped element.

Lumped or discrete FRAs use a fiber medium that is localized before or after transmission to fully or partially compensate for the transmission loss. Unlike a distributed FRA, all of the pump power is confined to the lumped element (see Figure 5). In this particular case, counterpropagating pump power is confined within the unit by the use of isolators surrounding the amplifier. Compared with Figure 3, no pump power enters the transmission line.

In discrete FRAs, the used fiber is a lot shorter (order tens instead of hundreds of km) compared to distributed FRAs. Since gains accumulate with fiber length, Raman amplification better fits in the distribuited FRAs class. Therefore, discrete FRAs are primarily used to increase the capacity of fiber-optic networks, opening up new wavelength windows, which are inaccessible by EDFAs. In FRAs, the signal is amplified at a wavelegth given by the frequency difference between the pump and the Stokes frequencies, thus by choosing the pump wavelength, gain at any wavelength can be obtained. Moreover, by combining multiple pump wavelengths, the shaping of the gain spectrum can be achieved, whereas by combining several pumps centered at different wavelengths, a flat gain in an ultrawide bandwidth is allowed.

**Figure 6:**
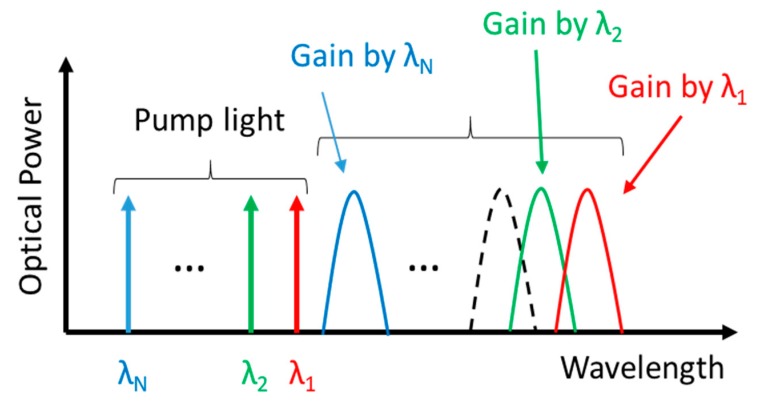
Multi-wavelength pumping technique for wideband Raman amplifiers.

The latter issue, i.e., a flat gain, is highly desirable in WDM systems in order to equally amplify all the channels. For this reason, flat gain of broad-band Raman amplifier, is considered one of the most important goal for the optimization of fiber communications. This task can be obtained by simultaneously using pumps at different wavelegths that generate Raman gain lightly shifted from each other so as to partly overlap each other leading to a composite gain (see Figure 6). The final Raman gain can be very flat over a wide range of wavelegth by accurately designing each pump wavelength and power. The gain-band expansion that can be achieved by multi-wavelength pumping is determined by the magnitude of the Stokes shift. A large Stokes shift is therefore desirable for a wideband FRAs using multi-wavelength pumping [55]. The maximum amplification bandwidth of a FRA is about 100 nm for silica fiber in the 1550-nm band, so the composite gain could cover a nearly 100-nm signal band [56]. They are particularly useful for amplifying (dense) WDM systems.

Using a broadband pumping approach, amplifiers with gain bandwidths greater than 100 nm have been demonstrated [57] with average net gain of 2 dB gain for SMF, while for dispersion-shifted fiber and reverse dispersion fiber the net gain was of 6.5 dB. A 12- wavelength-channel WDM LD (high-power laser diode (LD) pumping unit), with a range of wavelength from 1405 to 1510 nm and combined by an Il-MZI-integrated, using a planar lightwave circuit technology, were used to enlarge the gain bandwidth of Raman amplifiers [57] However, due to a strong interaction between pumps, these broadband FRAs typically need of more power at the shortest wavelengths pumps which amplify the longer wavelegth pumps, leading to at an increase of the noise properties.

Dispersion-compensating Raman amplifiers integrate two crucial tasks, dispersion compensation and discrete Raman amplification, into a single component [58,59,60]. Dispersion compensating fiber (DCF) devices, based on typical dispersion for the propagating mode, are usually in the order of several kilometers and, as a result, show important insertion loss. Hansen et al. demonstrated that Raman gain produced in the DCF was as a simple and attractive method to achieve lossless DCF modules, without affect system performance [58]. Due to a small mode-field diameter, a very low pump powers are required, like those available from semiconductor laser diodes. The DCF shows a net gain higher than loss, resulting in a wider system margin and the possibility to introduce other elements such as optical add-drop multiplexers into the system. In Ref. [61] a polarization-combined four channel WDM 14xx nm laser diode unit was used as pumping light source to demonstrate a broadband Raman amplification in a DCF. A fully compensation of the losses in the DCF were obtained by means of the Raman gain over 50 nm bandwidth within +/− 0.5 dB variation.

## 4. Fiber Raman Lasers (FRLs)

FRLs are similar to ordinary lasers. They basically involve three key components: A low-effective-area and high-nonlinearity optical fiber for providing Raman gain, mirrors for feedback and components for isolation between the pump laser (usually a rare-earth-doped fiber laser) and the Raman resonator. A first analogy is that, in FRLs, lasing occurs when the Raman-active gain medium is placed inside a cavity, for example, between mirrors reflecting the first Stokes wavelength. A second analogy is that, in FRLs, the threshold power is obtained when Raman amplification during a round trip is as large as to compensate the cavity losses. However, there are also some important differences between FRLs and traditional lasers. A first one is that an amplifier medium based on Raman gain is used rather than on stimulated emission from excited atoms or ions. A second difference is that the required wavelength for pumping Raman laser does not depend on the electronic structure of the medium, so it can be chosen to minimize absorption. In addition, it is worth highlighting the unique property of Raman laser, i.e., they can be operated at several wavelengths simultaneously. Higher-order Stokes wavelengths can be generated inside the active medium at high pump powers and they can be properly dispersed spatially in association with separate mirrors for each Stokes beam [62].

The first continuous-wave Raman laser in fiber was demonstrated in 1976 [63,64]. Pumping at the watts level, a distributed Raman gain, higher than the linear attenuation plus losses at splice/connector points, was induced in a single-mode glass fiber, placed inside a Fabry-Perot cavity. SRS process occurred in fiber resulting in an intense output, due to wavelength-selective feedback for the Stokes light of the Fabry-Perot cavity, which was formed by two partially reflecting mirrors. Higher-order Stokes wavelengths were generated inside the fiber at high pump powers, too. The spatial dispersion of various Stokes wavelengths through an intracavity prism allowed tuning of the laser wavelength.

**Figure 7:**
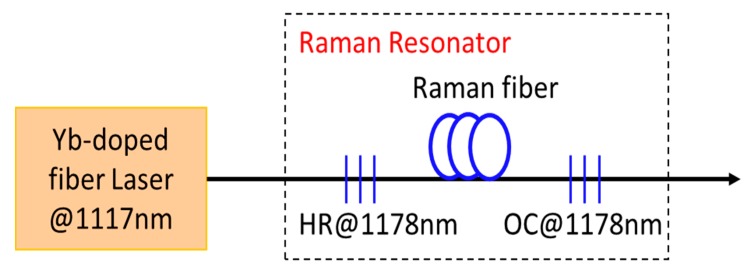
Schematic representation of a Raman fiber laser with one wavelength shift. (HR—high-reflectivity fiber Bragg grating, OC—output coupler—a low-reflectivity fiber Bragg grating).

A single wavelength shift FRL is a fiber resonator at the Stokes wavelength, in which SRS shifts the spectrum of the propagating pump radiation through an optical fiber towards lower frequency Stokes components. Raman lasing is obtainable in conventional single-mode telecom fibers (e.g., SMF-28) as well as other passive fibers, by trapping Stokes components by reflectors and by pumping the laser by a high-power rare-earth-doped fiber laser (commonly ytterbium). In an all-fiber configuration, enough positive feedback for lasing at the first Stokes wavelength is easily achieved. Directionality to the laser emission is provided by a high-reflecting fiber Bragg grating (reflectivity > 99%) on the input side and by a low-reflectivity output coupler (at Stokes wavelength) on the output side. Fiber Bragg gratings (FBGs) are narrow-band reflectors, acting as the mirrors of the laser cavity and inscribed directly into the core of the optical fiber used as the gain medium. However, we note that in Raman laser, when Raman gain of the active medium is very large, the laser can build up from noise to a substantial signal without any feedback. In addition, a single wavelength shift FRL, with a broadband flat Raman gain profile can be obtained using multiple pump wavelengths. Figure 7 shows a schematic diagram of a specific Raman fiber laser with one wavelength shift. In this particular case, the laser uses a 1117 nm ytterbium-doped fiber laser and converts it to 1178 nm.

**Figure 8:**
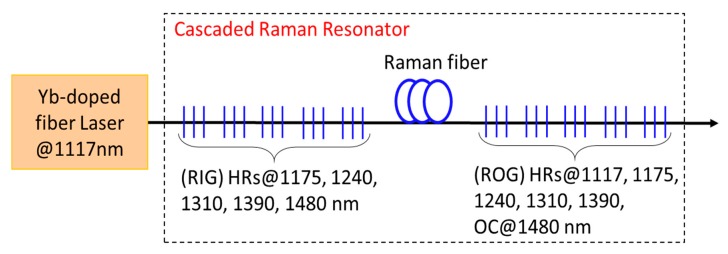
Schematic representation of a Raman fiber laser with multiple wavelength shifts by using a cascaded Raman resonator (RIG—Raman input grating set, ROG—Raman output grating set).

A multiple wavelength shifts FRL takes advantage of SRS cascading. The pump light gives rise to the “first-order” laser light in a single frequency-shifting step, which remains trapped in the laser resonator. Afterwards, the “first-order” laser light can be pushed to very high power levels becoming itself the pump for the generation of “second-order” laser light, which is shifted by the same vibrational frequency of first order. Using this technique, conversion of the pump light (typically around 1060 nm) to an “arbitrary” desired output wavelength through several discrete steps can be performed by using a single laser resonator. In Figure 8 is schematically reported the wavelength conversion over two or more Stokes shifts obtained by using a cascaded Raman resonator with fiber Bragg gratings incorporated in it, in order to realize nested cavities at each of the intermediate wavelengths. These intermediate wavelengths are chosen close to the peak of the Raman gain of the respective preceding wavelength; finally, a low-reflectivity output coupler terminates the wavelength conversion. The resulting output is at the designed final wavelength while only small fractions are at the intermediate wavelengths.

Tunability is an important property for lasers. The first tunable FRL, using a linear cavity configuration, was reported by Jain et al. [65]. A tuning range of 8 nm with an efficiency of 9% were demonstrated. However, the bulk optics (prisms and rotating mirrors), placed inside the laser cavity for tuning, introduced mechanical instabilities as well as coupling losses, which gave rise to high power thresholds and low efficiencies. In order to eliminate losses and to make a compact device, the approach based on bulk optics was replaced by an all-fiber design. In such a design, cavity mirrors were integrated within the fiber by the development of tunable fiber Bragg gratings [66]. In 1988, the first FRL based on fiber Bragg gratings was reported [67].

Since the Raman gain value of germanosilicate fiber is about eight times higher than for silicate fiber, these fibers are extensively used in FRLs. Due to their peak Raman shift of 440–490 cm^−1^, in an FRL with a 1064 nm pump source, the third and the sixth Stokes orders produce outputs at 1239 nm and 1484 nm, respectively. So, in order to make a FRL at 1484 nm, one would need six cascaded cavities. In Ref. [68], a GeO–doped high-power and widely tunable all-fiber Raman laser was demonstrated. The FRL, using a linear cavity configuration and a purely axial compression of the FBGs, was continuously tuned over 60 nm, from 1075 to 1135 nm. A Stokes output power up to 5 W, for 6.5 W of launched pump power was provided. A high efficiency over the whole tuning range, from 76.1 to 93.1% and laser thresholds varying from 0.78 to 2.59W were obtained, respectively.

It is worth noting that the peak Raman shift of low-loss phosphosilicate (P_2_O_5_-SiO_2_) fiber is 1330 cm^−1^, so when they are pumped by a 1064 nm pump source, the first Stokes (S1) and the second Stokes (S2) orders occur at 1239 nm and 1484 nm, respectively. This means that to make a FRL at 1484 nm, only two cascaded cavities would be required, thereby greatly increasing the FRL efficiency. In Ref. [69], a CW high-power Raman flber laser was reported, which was formed by a CW 8.4 W/ 1064 nm Yb-doped double-clad fiber laser as a pump, a 700 m phosphosilicate fiber and cascaded cavities with two pairs of FBG mirrors for the first and the second Stokes orders. A maximum output power of 2.24 W and slope efficiency of 32.8% at 1484 nm were demonstrated. In Ref. [70], Babin et al. demonstrated a simple all-fiber widely tunable phosphosilicate FRL of high efficiency. The laser had more than 50 nm tuning range and generated up to 3.2 W of output power with 72% maximum slope efficiency. The output power was almost constant in the range 1258–1303 nm.

Typically, the operating wavelengths of Raman laser are in the 1–1.5 µm region of the spectrum and have been created using silica glass as the base material with the desired application directed toward telecommunications. However, there are a large number of applications requiring high power laser with wavelengths longer than 2 µm. As a result of the versatility offered by FRLs, these types of sources are attractive for this purpose [71]. Chalcogenide glass fibers have a Raman gain coefficients approximately two orders of magnitude greater than the gain coefficients of silica. For wavelengths longer than 1.8 µm, the development of Raman fiber lasers based on chalcogenide glasses has now become technically achievable [72]. In Ref. [73] a Raman fiber laser, using a nonoxide glass as the gain medium and a 2 µm fiber laser pump source was demonstrated. Due to the low optical losses and high nonlinear index of commercially available glassy As_2_Se_3_ fiber, a combined output of 1 W at the first and second Stokes wavelengths of 2062 and 2074 nm, were generated respectively.

Single-frequency laser sources have a great number of applications, but are difficult to implement, suffer from poor robustness, poor quality (linewidth and stability) and are expensive. In recent years, there has been much interest in rare-earth doped distributed feedback (DFB) fiber lasers, due to their very low noise and high efficiency [74]. The oscillating wavelength can be accurately defined by a Bragg grating written directly into the fiber and inserting a π phase-shift (PS) at, or close, to the center of the Bragg grating structure. Of course, the operation of π-PS-DFB fiber lasers has been mostly limited to the spectral bands of rare-earth dopants [75].

A Raman gain based DFB fiber laser would have a number of potential advantages. First, the possibility of generating narrow linewidth low-noise oscillation in wavelength bands outside the one of rare-earth doped materials. Second, Raman based fiber laser systems do not suffer from issues associated with high-concentration rare-earth doped fibers, which limit their efficiency due to thermal effects. Using Raman gain in DFB fiber lasers was proposed for uniform FBGs by Perlin and Winful [76] for the first time, while a Raman DFB fiber laser based on a relatively short (<20cm), π-shift fiber Bragg grating were proposed by Hu et al. [77]. Westbrook et al. have realized the first functional DFB using the Raman gain in fiber, but with a very high threshold (40 W) was observed at 1584 nm. The authors showed that watt-level thresholds can be achieved by using a small-core high-numerical aperture (NA) fiber [78]. Shi et al. demonstrated that a polarized pump and an ultralong FBG (30 cm) could yield sub-watt threshold at a lasing wavelength of 1120 nm [79].

In reference [80] highly efficient Raman DFB fiber lasers with CW output power up to 1.6 W was demonstrated, for the first time. The DFB Bragg gratings were written directly into two types of commercially available passive germano-silica fibers. Two lasers of 30 cm length were pumped with CW power at 1068 nm up to 15 W. The threshold power was ~2 W for a Raman-DFB laser written in standard low-NA fiber, and only ~1 W for a laser written in a high-NA fiber, both of which oscillate in a narrow linewidth at ~1117 nm and ~1109 nm, respectively. The slope efficiencies were ~74% and ~93% with respect to absorbed pump power in the low-NA fiber and high-NA fiber respectively. Recently a Raman-DFB with lower thresholds at 1120 nm and 1178 nm was demonstrated [81]. Despite these developments, the performance in terms of slope efficiency has remained poor (1–7% for single-ended output) compared to the expected simulated efficiencies (40–50%).

By exploiting the multiple scattering of photons in a disordered gain medium, a random laser can be obtained allowing a coherent light source without a traditional cavity [82]. For different scientific and medical applications, ultrafast Raman fiber lasers are an interesting option. An efficient way to obtain a high power ultrafast Raman fiber laser is pulse pumping, but it requires a real-time synchronization between the pump pulses and laser cavity. In order to overcome this limitation, recently, random fiber lasers with distributed Raman gain and Rayleigh feedback in standard telecommunication optical fibers were demonstrated [83,84]. Their advantages are a simple and flexible design, quasi-CW operation, narrow spectrum generation, high beam quality and pump energy conversion efficiencies comparable to the ones of conventional cavity lasers [85]. In recent years, many works have been reported on the mechanism [84,85], the improvement of performance including power scaling [86,87], wavelength tuning [88,89], and Q-switching [90,91]. Nowadays, random fiber is a unique laser technology with various advantages in the fields of fiber sensing and optical communications [92,93].

A promising approach for the further development of devices based on SRS is represented by Photonic crystal fiber (PCF). The most important advantage of this technology is the reduction of the fiber length and the power levels required. The first preliminary results in photonic crystal fiber for Raman amplification and for an all optical Raman modulator was presented by Yusoff et al. [94]. An analysis of the Raman properties in triangular photonic crystal fiber was reported by Fuochi et al. [95], which investigated the influence of presence of the germanium doped core on the Raman properties. A model of PCF amplifiers was developed by Bottaccini et al. [96], which demonstrated that germanium doped PCF can enhance the gain for the same pump power. Finally, SRS in ethanol core PCF was demonstrated by Yiou et al. [97]. The approach of PCF with liquid could open new perspectives for SRS nonlinear properties with applications in optical sensing.

## 5. Raman Soliton Laser

Nonlinear effects are usually favored when pulsed operation are used, since high peak powers can be achieved with modest average powers. During their propagation inside optical fibers, short optical pulses (less than 10 ps) are affected by combination of chromatic dispersion and by the nonlinear effects [2,3,4], giving rise to a variety of phenomena, such as supercontinuum generation and soliton formation.

In the simplest situation, a single input pulse at the carrier frequency *ω*_0_ excites a single mode of the fiber. Each spectral component of the input field propagates as a plane wave and acquires a slightly different phase shift because of the frequency dependence of the propagation constant β*ω*. For this reason, it is useful to expand β*ω* in a Taylor series around the carrier frequency *ω*_0_, and, depending on the pulse bandwidth, the second-order dispersion term (group velocity dispersion (GVD)) can be considered or third- or higher-order dispersion terms can be included. The GVD parameter (called β_2_) can be positive or negative with values in the range of 0.1–20 ps^2^/km, depending on how close the pulse wavelength is to the zero-dispersion wavelength of the fiber. When the dispersive term can be neglected, spectral changes induced by SPM are a direct consequence of the time dependence of nonlinear phase shift. The time dependence of δ*ω* is referred to as frequency chirping. The chirp induced by SPM increases in magnitude with the propagated distance. In other words, new frequency components are generated continuously as the pulse propagates down the fiber. The situation changes drastically when both the GVD and SPM are equally important and must be considered simultaneously inside the fiber. In the case of normal dispersion (β_2_ > 0), both the pulse shape and spectrum change as the pulse propagates through the fiber and the combined effects of GVD and SPM can be used for pulse compression.

An interesting situation occurs for anomalous dispersion (β_2_ < 0), where the generation of optical solitons can be obtained. Solitons are special types of optical wavepackets formed by the balance between nonlinearity (positive SPM) and dispersion (and/or diffraction). This could be surprising, since GVD affects the pulse in the time domain while the SPM effect is in the frequency domain. However, a small time-dependent phase shift added to a Fourier transform-limited pulse does not change the spectrum to first-order. If this phase shift is cancelled by GVD in the same fiber, the pulse does not change its shape or its spectrum as it propagates. In the context of optical fibers, the use of solitons for optical communications was, for the first time, suggested in 1973 [98] and observed in an experiment in 1980 [99]. Temporal solitons have recently been observed in optical fiber waveguides [98,99,100] in laser resonators [101], and in dielectric microcavities [102]. In each of these cases nonlinear compensation of GVD is provided by the Kerr effect.

**Figure 9:**
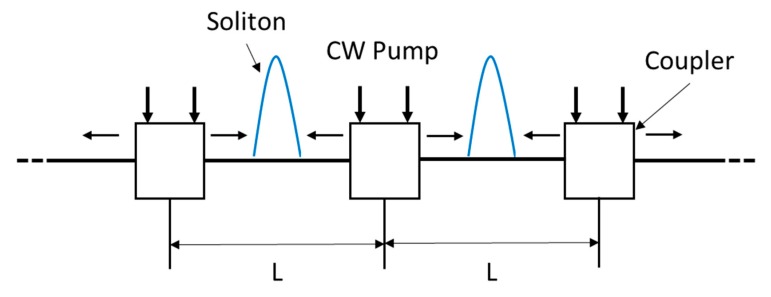
Schematic illustration of a soliton communication system. Solitons are amplified through SRS by injecting continuous wave (CW) pump radiation periodically.

Optical communication systems are often limited by fiber dispersion that broadens the pulse and by fiber losses. The use of fundamental solitons as an information bit solves the prolem of dispersion, because in a fiber channel, nonlinear phase modulation can compensate for linear group dispersion leading to pulses that propagate without changing temporal shape and spectrum. This mutual compensation of dispersion and nonlinear effects takes place continuously with the distance in the case of “classical” solitons and periodically with the so-called dispersion map length in the case of dispersion-managed solitons. Due to the fiber losses, soliton width begins to increase because of a decrease in the peak power during propagation inside the fiber, so amplification is required in order to recover original width and peak power. A proposed scheme makes use of SRS [103,104,105,106]. Figure 9 showes the basic idea. Solitons are launched into a fiber link consisting of many segments of length L. At the end of each segment, pump lasers inject CW light, upshifted in frequency from the soliton carrier frequency by about 13 THz, in both directions through wavelength dependent directional couplers. Since the Raman gain is distributed over the entire fiber length, the soliton can be adiabatically amplified.

During their propagation in optical fiber, soliton pulses are affected by SRS. The Intrapulse Stimulated Raman Scattering is a phenomenon that appears for short pulses with a relatively wide spectrum, in which Raman gain can amplify the low-frequency components of a pulse by transferring energy from the high-frequency components of the same pulse. As a result, the pulse spectrum shifts continuously toward the red side as the pulse propagates through the fiber [107,108]. This shift, called Raman-induced frequency shift (RIFS), was experimentally observed in 1986 [109], its Raman origin was also pointed out soon after [110], while a more general theory was developed later [7]. With the advent of microstructured fibers, much larger values of the RIFS (>50THz) were observed [111]. The RIFS is useful to generate Raman solitons whose carrier wavelength can be tuned by changing fiber length or input peak power. The effects of intrapulse Raman scattering can be dramatic in the context of solitons, where they lead to new phenomena such as decay and self-frequency shift of solitons.

Besides the Kerr nonlinearity, a secondary effect associated with soliton propagation is caused by Raman interaction. Equations describing SRS can be solved by inverse scattering methods and are found to have solutions. The so called Raman soliton was experimentally observed for the first time in 1983 [112]. When the wavelength of the pump pulse is close to or inside the anomalous dispersion region of an optical fiber, the Raman pulse should experience the soliton effects, i.e., under suitable conditions, almost all of the pump-pulse energy can be transferred to a Raman pulse that propagates undistorted as a fundamental soliton. We note that the soliton pulse is a bright soliton, while the Stokes pulse is a dark soliton and it has been demonstrated that quantum fluctuaction can induce them [113,114]. Raman solitons have also been generated by using a conventional fiber with the zero-dispersion wavelength near 1.3 µm led to 100-fs Raman pulses near 1.4 µm [115].

**Figure 10:**
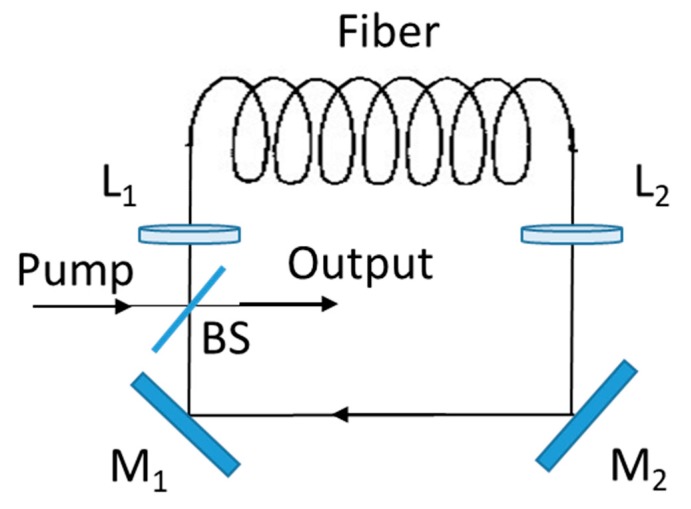
Schematic representation of the ring-cavity geometry implemented for Raman soliton lasers. BS is a dichroic beamsplitter, M_1_ and M_2_ are mirrors of 100% reflectivity, L_1_ and L_2_ are microscope objective lenses.

An interesting application of the soliton effects has led to the development of Raman soliton laser [116,117,118,119,120,121]. Such lasers provide their output in the form of solitons of widths 100 fs, but at a wavelength corresponding to the first-order Stokes wavelength., which can be tuned over a considerable range (10 nm). A ring-cavity configuration is commonly employed (see Figure 10). A dichroic beamsplitter, highly reflective at the pump wavelength and partially reflective at the Stokes wavelength, is used to couple pump pulses into the ring cavity and to provide the laser output. In a 1987 experimental demonstration of the Raman soliton laser [122], 10 ps pulses from a mode-locked color-center laser operating near 1.48 µm were used to pump the Raman laser synchronously. Even though Raman soliton lasers are capable of generating femtosecond solitons useful for many applications, they suffer from a noise problem that limits their usefulness [123]. The performance of Raman soliton lasers can be significantly improved if the Raman-induced frequency shift can somehow be suppressed [124].

## 6. Conclusions

In this paper the state of the art of fiber Raman amplifier and fiber Raman laser is described. Today, Raman amplifiers are widely commercialized devices. Almost every long-haul (typically defined 300 to 800 km) or ultralong-haul (typically defined above 800 km) fiber-optic transmission system uses Raman amplification. On the other hand, nowadays, commercially available fiber-based Raman lasers can deliver output powers in the range of a few tens of Watts in continuous-wave operation. In addition, due to their tunability, compactness, and capability for multi-wavelength operation, FRLs have great commercial potential in a variety of applications.
